# An Investigation of the Factors Reducing the Use of Hospital Services: A Systematic Review

**DOI:** 10.1002/hsr2.73220

**Published:** 2026-09-25

**Authors:** Sedighe Sadat Tabatabaei Far, Majid Alizadeh, Erfan Kharazmi, Zahra Kavosi

**Affiliations:** ^1^ Student Research Committee, School of Health Management and Information Sciences Shiraz University of Medical Sciences Shiraz Iran; ^2^ Health Human Resources Research Center, School of Health Management and Information Sciences Shiraz University of Medical Sciences Shiraz Iran

**Keywords:** hospital services, hospitalization, length of stay, reduction

## Abstract

**Background and Aims:**

Hospital service utilization has declined in many health systems due to policy shifts, limited resources, and alternative care approaches. This study aimed to systematically review the factors contributing to a reduction in the use or volume of hospital services.

**Methods:**

In this systematic review, articles published from 2018 to 2023 were reviewed by searching relevant keywords based on the PRISMA guidelines. The systematic search was conducted using English‐language texts related to the research question, “What are the factors reducing hospital services?” and electronic databases such as PubMed, Scopus, Science Direct, ProQuest, and Web of Science. The CASP checklist was used to evaluate the methodological quality of the included studies. Two researchers performed all the stages; finally, the selected articles were summarized in tables.

**Results:**

The database search yielded a total of 3705 articles, of which 30 were included in the review. The results of extracting the findings from the articles indicated four main concepts of factors reducing hospital services: “therapeutic factors,” “administrative‐support factors,” “preventive factors,” and “technological factors.” The studies assessed the impact of medications, treatment pathways, guidelines and standards, and treatment methods as effective therapeutic factors. Regulations were considered administrative or support factors, while health measures to control the spread of COVID‐19 and various vaccines were shown as preventive factors. Additionally, remote care and the use of new tools were categorized as technological factors influencing the reduction of hospital services.

**Conclusion:**

Therapeutic factors such as medications, managerial factors like regulations, preventive factors like vaccines, and factors related to new tools such as artificial intelligence were shown to be among the factors which reduce the use of hospital services. Therefore, if the focus on reducing services is hospital‐centered, these factors should be considered in policy‐making related to health service delivery.

## 1 Introduction

Healthcare is one of the fundamental needs of any society [1]. Today, changes in lifestyle, cultural and social structure, medical needs, and rapid population growth have posed significant challenges and obstacles to the provision of healthcare services. The rapid and ever‐increasing cost of healthcare services has reached a point where controlling these costs is the main problem for health systems in various countries, even the world's wealthiest nations [2]. Since paying attention to health and treatment and investing in this field increases labor productivity and production, allocating sufficient resources and utilizing them optimally in this sector is of great importance [1]. With rising healthcare costs, health policy‐makers focus on the most expensive sector of services, namely hospital services. Hospital services account for nearly half of the total healthcare costs. Therefore, improving the efficiency of these services by reducing costs and utilizing the potential capacities of healthcare institutions is deemed necessary [3, 4].

Hospitals are the main centers for providing health services, focusing on secondary treatment and prevention, which have undergone significant changes over time [5]. Changes in community needs and health system policies have played a major role in these transformations [6]. Among these changes, the emphasis of the World Health Organization on balancing prevention levels and providing community‐based services can be highlighted [7]. Community and family‐centered services are considered one of the fundamental changes in the overall health system policies; in some countries, health services are almost equally distributed between hospitals and the community [8]. For example, the establishment of counseling centers and nursing services has led to the development of the community‐oriented role of nursing [9].

Optimal use of available resources is one of the challenges for all hospital managers [10]. On the other hand, unnecessary hospitalization days increase the costs, decrease available resources for critically ill patients, and expose patients to hospital‐acquired infections [11]. Moreover, unjustified hospital stays have multiple social implications, including deactivating the workforce, causing emotional and psychological damage to families, and reducing the efficiency of inpatient units [12].

Various programs worldwide are being implemented to reduce the misuse of hospital beds. One such program is the establishment and use of day hospitals. Day hospitals introduce new diagnostic and therapeutic service lines beyond traditional patient care. They provide a middle tier of care between outpatient and inpatient attention, primarily serving patients in a stage of recovery who do not need acute care [13].

Various economic incentives have been used to reduce hospital costs. Although these incentives decrease the costs and hospital service utilization, they have not achieved optimal results regarding patient access to hospital services and the quality of provided services [3]. Studies conducted in different countries indicate that hospital care can sometimes be unnecessary and inappropriate. Unjustified hospitalization not only impacts the patients' health but also has economic and social consequences. Eliminating improper use of hospital services is one way to limit healthcare costs without compromising service quality [1].

In Canada, service stratification policies and a stronger emphasis on primary care have led to a decrease in direct hospital visits [14]. In Germany, economic pressures and the need for greater efficiency have shifted some hospital services to specialized outpatient centers and clinics [15]. A study by Mahmoodpour‐Azari et al. (2023) demonstrated that following the outbreak of COVID‐19, the utilization of both inpatient and outpatient services in Iranian hospitals and clinics declined significantly. This reduction was mainly attributed to changes in patient behavior, fear of contracting the virus, and modifications in health service delivery policies [16].

Given the changes in behavioral patterns regarding hospital service use, it is assumed that pandemics can impact the supply of medical services, people's willingness to seek care, the incidence of specific diseases, and thus hospital performance indicators [17]. For example, after the COVID‐19 pandemic was declared, various non‐pharmaceutical interventions were implemented in all countries to reduce pandemic effects, such as the temporary closure of non‐essential businesses and quarantine restrictions. These interventions posed a new challenge for health systems during the pandemic, impacting hospital service utilization [18] and leading to the postponement of many elective and non‐essential outpatient hospital services [19]. Meanwhile, hospitals were under pressure to respond to COVID‐19 cases and had to re‐prioritize their care services [20]. Due to the need for intensive care units for COVID‐19 patients, hospitals reorganized resources for COVID‐19 care units [21, 22, 23], and elective care units were nearly shut down. Consequently, hospital service utilization decreased to the extent that potential hospital revenues declined, particularly for hospitals heavily reliant on revenues from outpatient and elective services [24, 25].

Therefore, given the above‐mentioned points and the importance of reducing hospital costs, this study aimed to examine the factors that lead to a reduction in the use or volume of hospital services using a systematic review.

## 2 Methods

This study is a systematic review conducted according to Preferred Reporting Item for Systematic Reviews and Meta‐analyses (PRISMA) guidelines.

### 2.1 Research Question and Focus

The primary research question guiding this systematic review was: “What are the factors leading to a measurable reduction in the use or volume of hospital services?” While our initial focus was conceptualized around direct supply‐side constraints, our inclusive search strategy and eligibility criteria captured a broader range of evidence. Therefore, this review synthesizes factors that reduce hospital utilization, categorizing them by their primary mechanism:

1. Direct supply‐side factors: actions that limit the availability or provision of services (e.g., department closures, policy‐driven service reallocations, bed reductions).
2. Upstream demand‐side factors: interventions that reduce the clinical need for hospitalization (e.g., effective therapeutics, preventive vaccines, improved outpatient care), thereby decreasing patient demand for inpatient services.

Studies focusing solely on reducing the length of stay without affecting admission rates or service volumes were excluded, as the core outcome of interest is the reduction in service entry or overall volume.

### 2.2 Search Strategy

A comprehensive search was conducted across five international databases—PubMed, Scopus, ScienceDirect, Web of Science, and ProQuest—from 2018 up to November 19, 2023. The search encompassed peer‐reviewed articles published in both English and Persian. The search strategy incorporated combinations of keywords such as “hospital services,” “inpatient services,” “hospitalization,” and “policy,” utilizing Boolean operators (AND/OR) within the Title, Abstract, and Keywords fields. Additionally, MeSH terms were employed in PubMed to enhance the precision of the search outcomes (refer to Tables 1 and 2 for further details).

**Table 1:** Keywords used and their combination.

Concept category	Concept 2 (#2)	Concept 1 (#1)	Concept category
	Trend	Reduce	Hospital service
	Factor	Limit	Inpatient service
	Determinant	Decrease	Hospitalization
	Agent	Decline	
	Driver		
	Component		
	Indicator		
	Variable		
	Cause		
	Policy		
	#1: Trend* OR factor* OR determinant* OR agent* OR driver* OR Component* OR indicator* OR Variable* OR cause* OR polic*	#2: Reduc* OR limit* OR Decrease OR decline*	#3: “Hospital service*” OR “inpatient service*” OR hospitalization
Keyword combination	#1 AND #2 AND #3: (trend* OR factor* OR determinant* OR agent* OR driver* OR Component* OR indicator* OR Variable* OR cause* OR polic*) AND (reduc* OR limit* OR Decrease OR decline*) AND (“hospital service*” OR “Inpatient service*” OR hospitalization)		

**Table 2:** Search strategies used in various databases.

	Database	Search strategy	Time range	Search section	Language	Other database limitations	Number of articles found
1	PubMed	(Trend* OR factor* OR determinant* OR agent* OR driver* OR component* OR indicator* OR variable* OR cause* OR policy) AND (reduc* OR limit* OR decrease OR decline) AND (“hospital service” OR “inpatient service” OR hospitalization)	2018–2023	Title/abstract	—	Article type limited to: clinical trial, randomized controlled trial, meta‐analysis	1549
2	Science Direct	(Trend OR factor OR cause) AND (reduc OR decrease) AND (“hospital service” OR “inpatient service” OR hospitalization)	2018–2023	Title/abstract/keywords	English	Article type includes: research articles, case reports, practice guidelines, other	1729
3	ProQuest	(Trend* OR factor* OR determinant* OR agent* OR driver* OR component* OR indicator* OR variable* OR cause* OR policy) AND (reduc* OR limit* OR decrease OR decline) AND (“hospital service” OR “inpatient service” OR hospitalization)	January 1, 2018–December 20, 2023	—	English	Exclude document type: feature, report, commentary, correspondence, editorial, literature review, conference proceeding, review, letter to the editor, dissertation/thesis	381
4	Web of Science	(Trend* OR factor* OR determinant* OR agent* OR driver* OR component* OR indicator* OR variable* OR cause* OR policy) AND (reduc* OR limit* OR decrease OR decline) AND (“hospital service” OR “inpatient service” OR hospitalization)	2018–2023	Title	English	Exclude document type: review article, editorial material, letter, meeting abstract	26
5	Scopus	(Trend* OR factor* OR determinant* OR agent* OR driver* OR component* OR indicator* OR variable* OR cause* OR policy) AND (reduc* OR limit* OR decrease OR decline) AND (“hospital service” OR “inpatient service” OR hospitalization)	2018–2023	Title	English/Persian	Exclude document type: review, letter, editorial, note, conference paper, short survey	20
Total							3705
Duplicates							80
Final							3625

Eligibility criteria:

- Published between 2018 and 2023
- Articles in English or Persian
- Full‐text availability
- Focused on factors contributing to the reduction in the use, volume, or demand of hospital services
- Presence of the mentioned keywords in the title, abstract, or keywords

Exclusion criteria:

- Studies focusing only on hospital stay length
- Review articles, commentaries, protocols, theses, editorials, conference abstracts, reports, unpublished articles (grey literature), guidelines
- Articles without full text

### 2.3 Screening and Selection

Duplicates were removed using EndNote X8. Title and abstract screening was conducted independently by two reviewers (Tabatabaei and Alizadeh) and verified by a third reviewer (Dr. Kavosi). Discrepancies were resolved through discussion. Eligible full‐text articles were assessed, and only those meeting the inclusion criteria were selected.

The quality of all included articles was evaluated using the CASP checklist by two assessors. Each included study was independently appraised by two reviewers using the relevant CASP checklist. A quality score was calculated as the percentage of “Yes” responses across all applicable items. Studies that achieved at least 70% of the maximum possible score were considered methodologically sound and retained in the review. This threshold was pre‐specified to strike a balance between ensuring adequate study quality and retaining a sufficient number of studies for meaningful synthesis.

The latest version of CASP includes 10 items to assess three main areas: findings, validity of the findings, and feasibility at the local level, each comprising different sub‐sections [26]. In the end, any disagreements between the two authors were resolved through discussion and referral to a third person as a judge. Citation and publication bias were also considered, and articles with high citation rates were carefully evaluated. All the steps mentioned were repeated twice.

### 2.4 Data Extraction

After thoroughly reviewing the selected articles, the required information was extracted based on a pre‐designed summary and data collection form. This form included the following sections: title, corresponding author, study objective, study population, sample, country, study period, study design, instruments, methodology, results, and conclusions. A summary form was completed for each included article. Two researchers independently reviewed all articles and analyzed the completed forms, and the findings were presented in a table. In cases of disagreement, a third researcher provided input to resolve the discrepancies. All forms were prepared using Microsoft Word 2016.

### 2.5 Ethics Statement

This was not a human‐subject study; therefore, neither the institutional review board approval nor the subjects' informed consent was required.

## 3 Results

**Figure 1:**
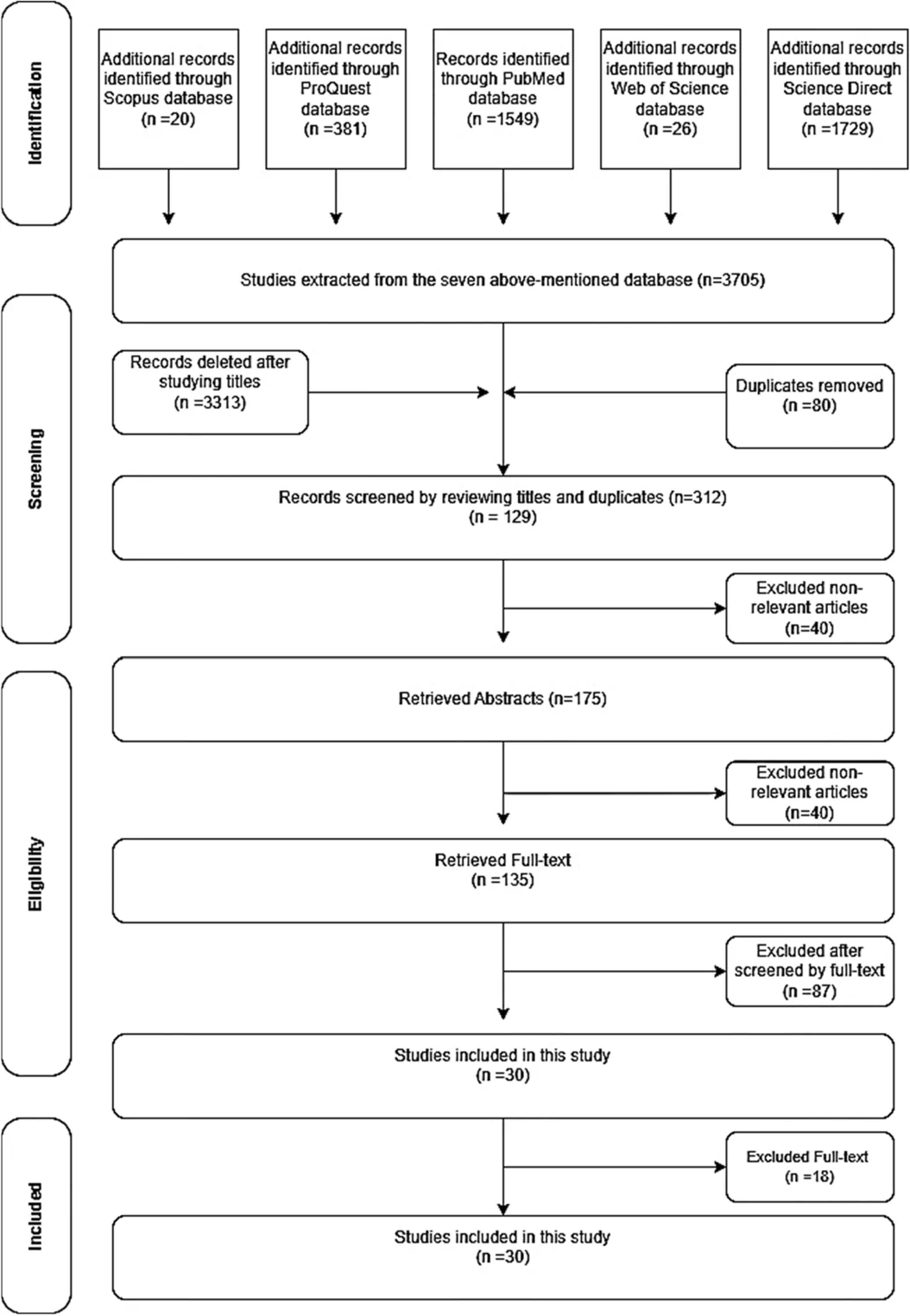
PRISMA flow diagram for the systematic review process.

**Figure 2:**
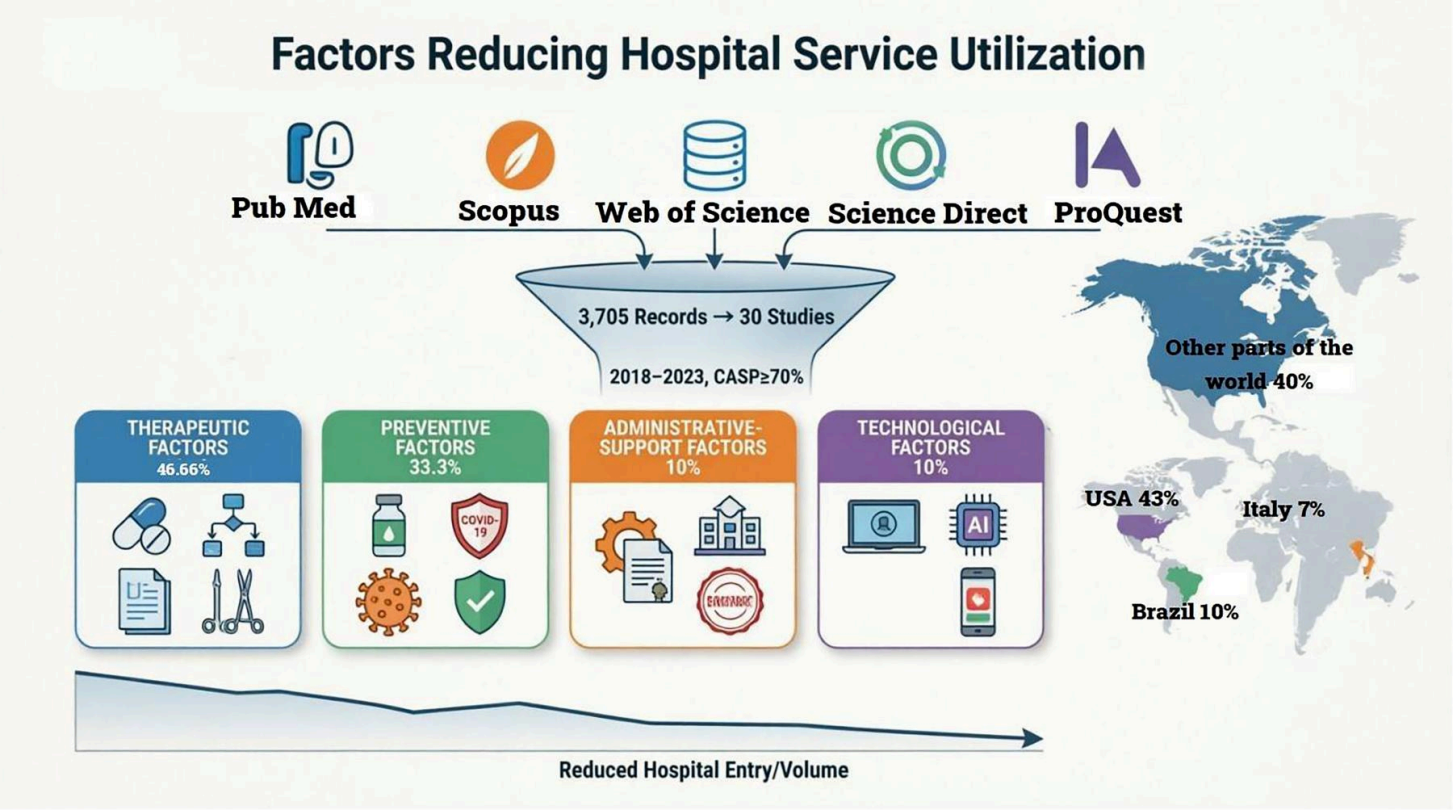
Graphical overview of the systematic review process and findings.

A total of 3705 references were identified through the search of electronic databases. Of these, 80 were duplicates. After screening the titles and abstracts, 3570 articles were excluded, and 135 full‐text articles were retrieved for further assessment. Finally, 48 articles matched our study aims; after the evaluation of the eligibility checklist was done, 30 articles received 70% of the relevant score (scoring at least 7 out of 10 items) and were included in the final synthesis. The detailed reasons for excluding the remaining 105 full‐text articles are presented in Table 3, while the overall study selection process is illustrated in Figure 1 (PRISMA flow diagram). All included articles were original research studies. The interventions identified to reduce hospital services comprised. The process of identifying and selecting studies is summarized in Figure 1. All articles were original research studies. The measures taken in the studies to reduce hospital services included therapeutic measures 14 (46.66%), administrative‐support measures 3 (10%), preventive measures 10 (33.33%), and technological measures 3 (10%). The majority of studies, 13 (43.33%), were conducted in the USA. The study designs included 7 (23.33%) cross‐sectional articles, 5 (16.67%) cohort studies, 5 (16.67%) retrospective studies, 5 (16.67%) clinical trials, 3 (10%) ecological studies, 3 (10%) interventional studies, 1 (3.33%) randomized controlled trial, and 1 (3.33%) quasi‐experimental study (Table 4 and Figure 2).

**Table 3:** Reasons for exclusion of full‐text articles and final inclusion during the systematic review selection process.

Phase/category	Reason for exclusion/outcome	Number of articles	Percentage^a^
Excluded articles			
Eligibility criteria	Studies focused only on length of stay without affecting admission rates/volumes; outcomes not aligned with study aims; population or setting mismatch	78	74.3%
Quality appraisal	Failed to meet the 70% threshold on CASP checklist (score < 7/10)	18	17.1%
Study design	Reviews, commentaries, protocols, theses, editorials, conference abstracts, or grey literature not identified during title/abstract screening	5	4.8%
Availability	Full text not accessible or retrievable despite extensive search efforts	4	3.8%
Subtotal excluded		105	100.0%
Included articles			
Final synthesis	Met all eligibility criteria and achieved the required CASP quality threshold (≥ 70%)	30	—
Total assessed	Full‐text articles assessed for eligibility	135	—

^a^ Percentages are calculated based on the total number of excluded full‐text articles (*n* = 105).

**Table 4:** Distribution of various factors and study types.

Variable	Categories	Subcategories	Number	Percentage
Factors	Therapeutic	Medication	14	46.66%
		Treatment pathways		
		Guidelines and standards		
		Treatment methods		
	Administrative‐support	Regulations	3	10%
	Preventive	Health measures to control COVID‐19	10	33.33%
		COVID‐19 vaccines		
		Rotavirus vaccines		
		Other vaccines		
	Technological	Telehealth	3	10%
		Use of modern tools		
Country	USA		13	43.33%
	International collaboration		4	13.33%
	Brazil		3	10%
	Italy		2	7%
	Australia		1	3.33%
	Canada		1	3.33%
	Sweden		1	3.33%
	Japan		1	3.33%
	Wales		1	3.33%
	Israel		1	3.33%
	Netherlands		1	3.33%
	Madagascar		1	3.33%
Study type	Cross‐sectional		7	23.33%
	Cohort		5	16.67%
	Retrospective		5	16.67%
	Clinical trial		5	16.67%
	Ecological		3	10%
	Intervention		3	10%
	Randomized controlled trial		1	3.33%
	Quasi‐experimental		1	3.33%

*Note:* The results extracted from the articles indicated four main categories of factors contributing to the reduction of hospital services—namely therapeutic, administrative‐support, preventive, and technological factors—which are summarized in Supporting Information S1: Table A1.

### 3.1 Therapeutic Agents

This category includes measures directly related to medical treatments and clinical care protocols that influence hospital service utilization. These factors are typically applied at the individual patient level and aim to reduce hospital admissions or readmissions by improving the effectiveness of care or introducing alternative treatment approaches.

#### 3.1.1 Drugs

Some studies have evaluated the effects of pharmacotherapy on reducing the use of hospital services among high‐risk patients with COVID‐19. In a study conducted in Wales, 2040 individuals received treatment with molnupiravir (359, 17.6%), nirmatrelvir‐ritonavir (602, 29.5%), or sotrovimab (1079, 52.9%). A lower risk of hospitalization or death at any point within 28 days was observed in treated participants compared to those who did not receive treatment [27]. In an international collaboration, compared to a placebo, dapagliflozin reduced the risk of first hospitalization and hospital admissions for all causes or death. Dapagliflozin reduced admission rates due to cardiac disorders, renal and urinary disorders, metabolic and nutritional disorders, and neoplasms [28]. Similarly, in another international collaboration with a total of 16,629 hospital admissions and 726 deaths, 331 fewer admissions and 58 fewer deaths with alirocumab were observed for patients with acute coronary syndrome compared to a placebo. This translates to 15.6 fewer total hospital admissions or deaths avoided with alirocumab per 1000 patients in a year of designated treatment. Alirocumab reduced overall hospital admissions and increased days alive in and out of the hospital compared to a placebo by reducing the days dead. Additionally, patients randomized to alirocumab had a higher likelihood of survival without hospitalization until the end of the study [29].

#### 3.1.2 Treatment Pathways

Some studies have described the methods of care. In Japan, an innovative clinical pathway shortened the length of hospital stay and prevented readmissions in patients with acute decompensated heart failure (ADHF). The clinical pathway included: (a) use of tolvaptan (7.5 mg/day) for the first 3 days as standard diuretics therapy for patients without hypernatremia (>145 mEq/L) who showed poor response to loop diuretics, (b) use of oxygen therapy for the first 3 days only when transcutaneous oxygen saturation is not maintained at 90% or higher, (c) use of intravenous fluid administration for the first 2 days only when other medications, including diuretics, were administered intravenously, (d) use of a urinary catheter for the first 2 days only when the patient could not go to the toilet alone due to dyspnea or low activity of daily living, (e) initiation of cardiac rehabilitation on the second day after admission unless the patient refused it. All these therapeutic interventions were automatically ordered. The length of hospital stay for patients in the clinical pathway group was significantly shorter than for patients in the non‐CP group, without an increase in in‐hospital mortality or readmission rates due to hospital stay duration within 30 days. The use of the clinical pathway was an independent negative factor contributing to the length of hospital stay for patients with ADHF, even after adjusting for other factors, including the use of tolvaptan. The clinical pathway significantly reduced the ratio of patients readmitted to hospitals due to ADHF within 6 months after discharge compared to the ratio in the non‐CP group [30]. Additionally, in the USA, the implementation of a safe cardiac pathway intervention reduced hospital admissions within 1 year for patients with acute chest pain. The heart pathway identified 30.7% of patients as low risk. Hospital admissions within 1 year decreased by 7.0% in the post‐implementation group compared to the pre‐implementation group. The implementation of the heart pathway was associated with reduced hospital admissions and low rates of adverse events in low‐risk patients during a 1‐year follow‐up [31].

A study conducted in the United States found that participants in an emergency department‐initiated patient navigation program (ED‐PN) showed a significant reduction in emergency department visits over a 12‐month evaluation period compared to usual care. The ED‐PN program targeted high emergency department utilizers who were insured through Medicaid. On average, patients had 1.4 fewer emergency department visits and 1.0 fewer hospital admissions per patient. Both groups increased their outpatient service utilization. ED‐PN patients showed a trend toward reduced hospital costs per patient, while Medicaid expenditures remained unchanged. Patients who demonstrated reduced emergency department use tended to be older and had lower health literacy [32]. Additionally, in another study in the USA, children enrolled in the in‐person navigation group reduced their risk of hospitalization over 12 months, making them 69% less likely to be hospitalized. In this randomized clinical trial assessing the effects of health care utilization of programs designed to address social needs among families, children enrolled in the navigation group were significantly less likely to be hospitalized post‐intervention; however, they were just as likely to visit the emergency department. Following the initial in‐person visit, navigation services included phone, email, and/or in‐person follow‐ups for up to 3 months [33]. Findings of the study from Western Australia showed that the initiation of community‐based palliative care before the last 6 months of life was associated with a lower average number of unplanned hospital admissions in the last 6 months of life. The associated costs were also lower compared to similar groups ($12,976 vs. $13,959) [34].

#### 3.1.3 Guidelines and Standards

In another study in Israel, adherence to guideline‐recommended medical therapy (GRMT) and the use of healthcare resources following an acute myocardial infarction (AMI) were investigated. A reduction in hospital admissions, emergency department visits, and costs was observed, along with an increase in primary clinics and consultation visits. Compliance with GRMT following AMI was associated with decreased mortality, hospital admissions, and costs [35]. In a study in Italy, the categories of medium and high adherence score (compared with the low category) were significantly associated with a lower risk of hospitalization. Among the adherence score indicators, the initial prescription of disease‐modifying antirheumatic drugs (DMARDs) showed the strongest positive effect, while the long‐term use of glucocorticoids had the worst negative effect. Consequently, in early rheumatoid arthritis, adherence to quality care standards was associated with a lower risk of hospitalization [36].

#### 3.1.4 Treatment Methods

In the USA, among new users of positive airway pressure (PAP) therapy, the rate of hospital admissions for COPD‐related conditions was significantly lower in the 1 year following the initiation of PAP therapy compared to the 1 year before initiation. However, emergency department visits (for any reason or COPD‐related conditions) and hospital admission rates for any cause did not significantly differ between the pre‐ and post‐initiation periods. PAP therapy was more beneficial in older individuals, those with higher COPD complexity, and those with three or more comorbidities. Initiation of PAP therapy in elderly patients with overlap syndrome was associated with reduced hospital admissions for COPD‐related conditions but not for all admissions and emergency department visits [37]. A study in the USA investigating the impact of endoscopy and colonoscopy interventions showed that, compared to non‐pregnant women with acute biliary pancreatitis, endoscopic retrograde cholangiopancreatography (ERCP) and cholecystectomy were less frequently performed during pregnancy. Weekend admissions and hospital stays longer than 1 week increased the risk of 30‐day readmission, while ERCP and cholecystectomy reduced the likelihood of early readmission during pregnancy [38]. In an international collaboration on the impact of intensive periodontal treatment compared to the control group, the adjusted hazard ratio for hospitalization in cardiovascular patients was 0.78 in the treatment group. The treatment group showed a significantly lower cumulative incidence of cardiovascular diseases and mortality. Intensive periodontal disease treatment was associated with reduced risks of cardiovascular diseases and overall mortality in patients undergoing hemodialysis [39]. A study in the USA on the impact of behavioral, physical, and psychosocial factors on reducing hospital services indicated that changes in certain health behaviors (e.g., individuals engaging in frequent physical activity); physical health conditions (e.g., individuals with cancer); and psychosocial factors (e.g., those who helped friends/neighbors/relatives for 100–199 h per year) were associated with reduced frequency and duration of overnight hospital stays. Notably, some psychosocial factors (such as informal help) had an impact equivalent to or greater than some physical health conditions (such as diabetes) and health behaviors (such as smoking) [40].

Cross‐context comparison: Therapeutic interventions were most frequently reported in studies from countries such as the United States, Japan, and European nations. Clinical infrastructure allowed for controlled trials and guideline adherence. However, the consistency of findings across various contexts suggests that treatment‐based strategies can universally contribute to reduced hospitalization when appropriately applied.

### 3.2 Administrative‐Support Factors

This category refers to system‐level policies, administrative regulations, and organizational frameworks that indirectly influence hospital service utilization. These measures are typically implemented at the policy or institutional level and aim to reduce preventable hospitalizations through structural and regulatory reform.

#### 3.2.1 Regulations

A study conducted in North Carolina on the impact of stay‐at‐home orders and other COVID‐19‐related policies on trauma hospitalizations and disparities in the United States revealed that statewide stay‐at‐home orders increased the risk of hospitalization for Black/African American residents, exacerbating pre‐existing disparities. After the public health emergency, especially among older individuals, hospitalizations due to unintentional injuries decreased, but they returned to 2019 levels within a few months. The study indicated that fear of COVID‐19 might have led to a reduction in unintentional hospitalizations, particularly among the elderly [41]. The Affordable Care Act (ACA), which aimed at increasing the number of insured individuals, may lead to sufficient primary care management and a reduction in preventable hospitalization rates. Findings from a study in the United States showed a significant decrease in preventable hospitalization rates following the implementation of the ACA, from 12.0% to 10.8%, and from 11.5% to 10.6% following the expansion of Medicaid. Bacterial pneumonia rates decreased from 1.5% to 0.6%, along with a reduction in chronic obstructive pulmonary disease (COPD) and asthma rates among older adults, from 1.9% to 1.7% post‐expansion [42]. Results from the Missouri Quality Initiative in the United States demonstrated 6‐year trends in reducing hospitalizations for nursing home residents. Full‐time advanced practice registered nurses and a centralized operations support team focused on reducing unnecessary hospitalizations for long‐term nursing home residents. The total number of transfers for the years 2014–2019 was 6913, and the average transfer rate per 1000 resident days decreased from 2.48 in 2014 to 1.89 in 2018, with a slight increase to 1.99 in 2019 [43].

Cross‐context comparison: Administrative‐support measures were predominantly reported in the United States, reflecting the country's active use of health policy reforms to manage service utilization. Such factors may be less documented or applied in lower‐resource settings, possibly due to weaker administrative infrastructures or lack of nationwide health coverage systems.

### 3.3 Preventive Factors

Preventive factors involve public health strategies, particularly immunization programs and infectious disease mitigation, that reduce hospital admissions by preventing the onset or worsening of illness within the population.

#### 3.3.1 Health Measures to Control the Spread of COVID‐19

A study in Brazil showed that in the years 2020 and 2021, the number of hospital admissions was significantly lower than time series forecasts. However, mortality rates in these years were three times higher compared to the previous year, and six times higher in COVID‐19 patients compared to those without the disease. Hospitalization costs in 2020 and 2021 were lower than in previous years. Health measures to control the spread of COVID‐19 led to a reduction in hospital admissions of children due to other respiratory infections in public hospitals in São Paulo, Brazil [44]. Additionally, the impact of COVID‐19 mitigation strategies, such as sanitation and mask use, on asthma hospitalizations in Brazil, led to a significant reduction in the average incidence of hospital admissions in 2020, with rates of 59% for ages 1–14 years, 37% for ages 20–59 years, and 60% for ages over 60 years. In 2020, hospital admissions due to asthma, especially among children and older groups, decreased during the COVID‐19 pandemic, which may be associated with a reduction in the incidence of many respiratory viral infections [45].

#### 3.3.2 COVID‐19 Vaccines

The results of a study in Canada showed that among adolescents, two doses of the mRNA vaccine were associated with an 85% lower likelihood of hospitalization among SARS‐CoV‐2 cases caused by the Omicron variant. Among children, one dose of the mRNA vaccine was associated with a 79% lower likelihood of hospitalization among SARS‐CoV‐2 cases caused by the Omicron variant. Despite immune escape by SARS‐CoV‐2 variants, vaccination remains associated with a lower likelihood of hospitalization among Omicron‐infected adolescents and children, even when vaccines do not prevent infection [46]. In another study conducted in the Netherlands, from January 6, 2021, to August 30, 2022, approximately 98,170 hospitalizations of COVID‐19 patients were prevented through vaccination. The estimated prevented hospitalizations were lowest among individuals aged 12–49 years and highest among those aged 70–79 years. Additionally, more admissions were prevented during the Delta period (72.3%) compared to the Omicron period (63.4%) [47]. In an international partnership using electronic health registries for individuals aged 80 and above, with the unvaccinated ones considered as a reference, the vaccine effectiveness against COVID‐19‐related hospitalizations decreased from 66.9% to 36.1% for initial vaccination and from 95.6% to 67.7% for the first booster. Similar trends were observed for individuals aged 65–79 years. The effectiveness of the second booster vaccine against hospitalizations ranged from 82.0% (95% CI: 75.9; 87.0) to 83.9% (95% CI: 77.7; 88.4) for those aged 80 and above, and from 39.3% to 80.6% for those aged 65–79 years. The effectiveness of the first booster vaccine against COVID‐19 mortality decreased over time for both age groups, while the effectiveness of the second booster against mortality remained above 80% for those aged over 80 years [48]. Additionally, a study conducted in the United States following the implementation of the SARS‐CoV‐2 vaccine prevented 721 hospitalizations (95% CI: 126–1241). The vaccination program reduced COVID‐19 hospitalizations among the population aged 65 and older, who were initially eligible, by approximately 15% in the first 8 weeks. Racial/ethnic groups and areas with higher vaccine coverage generally experienced greater reductions in RR estimates [49].

#### 3.3.3 Rotavirus Vaccine

Findings showed that in Sweden, the incidence rate of rotavirus, with a 95% confidence interval, for children under 5 years old who were hospitalized due to rotavirus gastroenteritis was 2.9 (2.8–3.1) per 1000 person‐years before vaccination and 0.65 (0.56–0.74) after vaccination, with a rate ratio of 0.22 (0.19–0.26). The rate of gastroenteritis due to all causes was 5.6 (5.4–5.9) before vaccination and 2.5 (2.3–2.7) after vaccination (RR 0.45 (0.42–0.50)). Among the patients under 5 years old, those with underlying conditions constituted a higher proportion after vaccination, compared to before vaccination (30.7% vs. 24.2%). Rotavirus vaccination reduced the number of children under 5 years old who needed hospital care for gastroenteritis [50]. Another study in Madagascar showed that hospitalizations due to diarrhea decreased after the introduction of the rotavirus vaccine. The average annual hospitalization rate for diarrhea was 26% (range: 31%–22%) before the vaccine introduction. This rate was 25% in the year of vaccine introduction, 17% in 2015, and 16% in 2016. Before the vaccine introduction, 56% of stool samples were positive for rotavirus. The positive percentage was 13% in 2015 and 12% in 2016 [51].

#### 3.3.4 Other Vaccines

A study in Brazil showed that hospitalizations decreased by 24.5% after the injection of the pneumococcal conjugate vaccine (PCV‐10). The monthly average hospitalizations decreased from 681 (2005–2009) to 514 (2011–2015). The hospitalization rate decreased from 56.1 per 1000 live births in the 5 years before PCV‐10 to 43.4 in the subsequent 5‐year period (a decrease of 22.7%). Comparing the values predicted by the SARIMA model for the scenario without PCV‐10 in the second 5‐year period with the values reported after the implementation of PCV‐10 showed that the estimated number of prevented hospitalizations was 8682 5 years following the vaccine introduction. Consequently, 5 years after implementing PCV‐10, hospitalizations of children with pneumonia in Pernambuco decreased by 22% [52]. Additionally, based on the findings of a study in Italy, the hospitalization rate for chickenpox showed a declining trend by age. Children under 1 year of age were the most affected age group in every region of Italy, while lower incidence rates were observed in older age groups. The hospitalization rate for varicella significantly decreased after the introduction of varicella vaccination. In the first 5 years following the introduction of vaccination, the hospitalization rate significantly decreased, especially among infants under 1 year old. The total percentage change in the under‐one and 1–5‐year age groups was −80.0% and −86.7%, respectively. Consequently, the hospitalization rate is related to both vaccination coverage and the number of years since the introduction of vaccination [53].

Cross‐context comparison: Preventive interventions were documented in countries, including Brazil, Canada, Sweden, and Madagascar. The global effectiveness of vaccinations suggests that public health strategies can reduce hospital service burden broadly, although their impact may vary depending on coverage, implementation quality, and population structure.

### 3.4 Technology Factors

This category includes the use of digital health technologies, telemedicine, and advanced analytics tools aimed at improving outpatient care, monitoring, and early intervention, thereby reducing the need for hospitalization.

#### 3.4.1 Telecare

Based on findings, health technology programs that include individualized and personalized planning for relapse prevention with treatments delivered via smartphones and computers, as well as web‐based decision support programs, can reduce hospitalizations in patients with schizophrenia. A study conducted in the United States with 438 patients showed that an advanced technology‐based relapse prevention program reduced hospital days by an average of 4 days per patient in the first 6 months compared to usual services. According to this study, relapse prevention through health technology may improve the care provided while simultaneously reducing hospitalization‐related costs [54]. Another study in the United States on the impact of school‐based telemedicine enhanced asthma management (SB‐TEAM) to overcome barriers to preventive asthma care found that among children in the SB‐TEAM group, 196 (98.0%) had one or more telehealth medical visits, and 165 (82.5%) received supervised treatment through the school. Children in the SB‐TEAM group increased their symptom‐free days every 2 weeks after the intervention compared to children in the usual care group, with the most significant difference observed at the final follow‐up. Additionally, children in the SB‐TEAM group were less likely to visit the emergency department or be hospitalized due to asthma [55].

#### 3.4.2 Use of Modern Tools

Another study conducted in the United States indicated that interdisciplinary team interventions with risk‐based machine learning models were associated with hospitalization rates and hospital days in hemodialysis patients compared to the control group. The use of machine learning models can predict the 12‐month risk of outpatient hemodialysis patients being hospitalized multiple times to guide personalized interdisciplinary interventions in the dialysis hospitalization reduction program [56].

Cross‐context comparison:

Technological strategies were predominantly reported from high‐income countries with well‐developed digital infrastructures. While their potential is high, such interventions require investments in IT systems, training, and access, which may limit their scalability in resource‐constrained settings.

## 4 Discussion

This study aimed to investigate the factors contributing to the reduction of hospital service utilization. Various researchers in their studies have referred to multiple factors, most of which being related to therapeutic factors, while others focus on administrative‐support, preventive, and technology‐related factors.

In the therapeutic category, the predominant factors were related to the impact of treatment pathways in reducing hospital service usage. Clinical pathways for cardiac care reduce hospital admissions. In one study, it was found that early decongestion with tolvaptan improved HF symptoms, leading to early discharge of patients from oxygen therapy, intravenous fluid infusion, and urinary catheters, subsequently enabling early initiation and enhancement of cardiac rehabilitation. The EVEREST trial also concluded that one of the largest randomized controlled trials globally, which evaluated the effect of tolvaptan on ADHF, showed that administering tolvaptan within 48 h of admission significantly improved heart failure symptoms, including dyspnea and edema. Additionally, the study by Ittiporn and Prajongdee revealed that the hospitalization rate for nearly half of the severe asthma cases in the group adhering to the clinical pathway was significantly lower. Accurate assessment of severity and adherence to the clinical pathway can reduce hospital admissions in children with severe asthma [57]. Most studies on the effects of healthcare interventions designed to address social needs among families found that children enrolled in guidance programs were significantly less likely to be hospitalized after the intervention, but they were equally likely to visit the emergency department. These findings enhance our understanding of the effects of addressing social needs in clinical settings as part of a comprehensive strategy to improve health and reduce healthcare utilization. However, in a study conducted on infants living in low‐income families in Boston, Sege et al. reached different conclusions. They randomly assigned families to a family support specialist who provided clinical education and social care support during clinic visits, routine home visits, and through phone, email, or text. Six months after enrollment, infants in the intervention group were less likely to visit the emergency department compared to the control group [58]. These differences in emergency department usage may reflect the added value of the clinical intervention component in the study by Sege et al.

In the administrative support category, regulations played a significant role in reducing hospital service utilization. For example, the Affordable Care Act (ACA) may lead to sufficient primary care management and a reduction in preventable hospitalization rates. Therefore, countries that have not implemented Medicaid expansion should prioritize it as it may lead to a reduction in preventable hospitalizations. Additionally, the rate of preventable hospitalizations may be considered a quality measure for assessing access to and effectiveness of primary care interventions.

In the preventive category, most factors were related to the impact of vaccines in reducing hospital service utilization. Vaccines, including COVID‐19, rotavirus, and others, were recognized as preventive agents for hospital admissions. One study found that rotavirus vaccination reduced the number of children under 5 years who needed hospital care for gastroenteritis. Recent studies from other Northern European countries have also demonstrated the sustained impact of rotavirus vaccination on hospital admissions for rotavirus gastroenteritis and all‐cause gastroenteritis [59, 60]. The reduction in outpatient cases treated for gastroenteritis was less pronounced compared to hospitalized patients, as rotavirus vaccines are more effective in preventing severe infections [61]. However, changes in emergency department protocols with frequent oral or intravenous hydration and increased use of ondansetron and racecadotril may have also contributed to the observed reduction in admissions for all‐cause gastroenteritis and rotavirus gastroenteritis noted several years before vaccination [50].

In the technology category, telecare was frequently mentioned. Telecare can be utilized as a technology‐related factor to reduce hospital admissions. It was found that relapse prevention with advanced technology is an effective and feasible way to reduce readmission days for patients with schizophrenia. Advanced technology‐based relapse prevention, tailored to each patient, can enhance recovery and the ability of individuals to remain in the community, significantly reducing the costs of managing schizophrenia and offsetting the costs of accessing the technology [54]. Additionally, previously reported high acceptance and satisfaction suggest that these patients are willing and able to engage in a new and technologically advanced approach to relapse prevention [62].

One of the main limitations of this study was the lack of access to the full text of some articles related to the topic.

It is recommended that these factors be tailored to the capacity and capabilities of healthcare organizations to reduce hospital service costs and patient length of stay. The cost‐effectiveness of using medications and new treatment methods should be evaluated in patient care processes. Managerial actions should be taken to predict pandemics and manage the patients in coordination with the healthcare staff. Vaccination programs should be pursued vigorously, and policies should be adopted to utilize modern technologies like artificial intelligence as supportive tools for patient management and care in healthcare settings.

## 5 Conclusion

The findings of this study highlight therapeutic factors such as medications, managerial factors such as regulations, preventive factors like vaccines, and factors related to modern tools such as artificial intelligence as significant contributors to reducing hospital service utilization. Consequently, the integration of these factors into policy‐making is deemed essential. Researchers hope that the results of this study can aid in identifying the factors that decrease hospital service usage and subsequently pave the way for effective planning to address hospital cost issues.

## Author Contributions

**Sedighe Sadat Tabatabaei Far:** investigation, formal analysis, data curation, resources, writing – original draft, writing – review and editing, project administration. **Majid Alizadeh:** investigation, writing – original draft, formal analysis, writing – review and editing. **Erfan Kharazmi:** conceptualization, methodology, supervision, writing – original draft, formal analysis, writing – review and editing. **Zahra Kavosi:** conceptualization, methodology, investigation, formal analysis, data curation, supervision, writing – original draft, writing – review and editing.

## Funding

The authors have nothing to report.

## Conflicts of Interest

The authors declare no conflicts of interest.

## Transparency Statement

Dr. Erfan Kharazmi affirms that this manuscript is an honest, accurate, and transparent account of the study being reported; that no important aspects of the study have been omitted; and that any discrepancies from the study as planned (and, if relevant, registered) have been explained.

## Supporting information


Supporting File

## Data Availability

The authors confirm that the data supporting the findings of this study are available within the article and its supplementary materials. Additional data or materials may be provided by the corresponding author upon reasonable request.
