# Supplementary material for: An Investigation of the Factors Reducing the Use of Hospital Services: A Systematic Review
Source: Health Sci Rep. 2026 Sep 25;9(10):e73220. doi: 10.1002/hsr2.73220 (PMC13615344; doi:10.1002/hsr2.73220)
Supplement: Supplementary file 1 [file HSR2-9-e73220-s001.pdf]

Table A1 Key Findings from Selected Studies								
**No.**	**Authors/Publication Year**	**Title**	**Study Population**	**Study Sample**	**Country**	**Study Type**	**Methodology**	**Main Results Summary**
1	Simons et al. (2023)	*Vaccine Effectiveness Against Hospitalization in Adolescents and Children with SARS-CoV-2 in Ontario, Canada: A Retrospective Cohort Study*	Canadian adolescents/children with SARS-CoV-2	62 hospitalized and 27,674 non-hospitalized SARS-CoV-2 cases (May 2021–Jan 2022)	Canada	Retrospective Cohort	Retrospective linkage of SARS-CoV-2 cases (Ontario Public Health data) with vaccination records (COVaxON). Multivariate logistic regression estimated vaccine effectiveness against hospitalization pre- and post-Omicron emergence.	Among adolescents, two mRNA vaccine doses were associated with **85% lower hospitalization odds** for Omicron-related SARS-CoV-2 cases. In children, one mRNA dose reduced hospitalization odds by **79%**. Sensitivity analyses (E-values) suggest unmeasured confounding is unlikely to explain these effects.

2	Di Mello Araújo et al. (2023)	*Impact of 10-Valent Pneumococcal Conjugate Vaccine on Childhood Pneumonia Hospitalizations*	Children with pneumonia in Brazil	Children with pneumonia in Brazil's Unified Health System	Brazil	Ecological Time-Series	Ecological time-series analysis comparing pneumonia hospitalizations (7 months–4 years) 5 years pre- (2005–2009) and post-PCV-10 (2011–2015). Descriptive statistics, chi-square, t-tests, and SARIMA forecasting.	Post-PCV-10 introduction, pneumonia hospitalizations decreased by **22.7%** (56.1 to 43.4 per 1,000 live births). SARIMA modeling estimated **8,682 hospitalizations prevented** over five years post-vaccination.
3	Evans et al. (2023)	*Real-World Effectiveness of Antivirals and Monoclonal Antibodies in Preventing COVID-19 Hospitalizations in Wales*	High-risk COVID-19 patients in Wales	7,013 high-risk non-hospitalized COVID-19 patients (Dec 2021–Apr 2022)	Wales	Retrospective Cohort	Retrospective cohort using anonymized EHRs from the SAIL Databank (Wales). Time-dependent Cox models adjusted for age, comorbidities, and vaccination status.	Treated high-risk COVID-19 patients had **35% lower risk** (95% CI: 18–49%) of hospitalization/death within 28 days vs. untreated. No significant difference between Molnupiravir, Nirmatrelvir-Ritonavir, or Sotrovimab.

4	Homan et al. (2023)	*Technology-Driven Relapse Prevention Reduces Hospitalizations in Schizophrenia*	Outpatients with schizophrenia in the USA	438 outpatients with schizophrenia (2013–2015)	USA	Quasi-Experimental Trial	Quasi-experimental trial in outpatient clinics. Participants received usual care or a 6-month tech-based relapse prevention program (smartphone apps, web tools).	A technology-enhanced relapse prevention program reduced psychiatric hospitalizations: **24 % intervention vs. 43% control** hospitalized over 6 months. Hospital days decreased by 5 days in the intervention group.
5	Van Iersel et al. (2023)	*COVID-19 Hospitalizations Prevented by Vaccination: Estimates for the Netherlands*	Vaccinated population in the Netherlands	Vaccinated individuals in the Netherlands	Netherlands	Cross-Sectional	Cross-sectional analysis using age-specific vaccine effectiveness (VE) and coverage (VC). Admissions causally linked to COVID-19 were excluded post-Jan 2022.	**98,170 hospitalizations prevented** by vaccination (Jan 2021–Aug 2022), representing **57–67% of projected admissions**. Highest impact in ages 70–79; greater reductions during Delta (72.3%) vs. Omicron (63.4%).

6	Kislaya et al. (2023)	*Monitoring COVID-19 Vaccine Effectiveness Against Hospitalization and Death in Older Adults*	Older adults in six European countries	Adults ≥65 years (Oct 2021–Nov 2022)	Multinational	Cross-Sectional	Multi-country EHR analysis (Belgium, Denmark, etc.). Monthly VE estimated using Cox models with 8-week follow-up periods.	Waning VE against hospitalization: primary series declined from **66.9% to 36.1%** (≥80 years). Second boosters maintained **82–84% effectiveness** (≥80 years). VE against death remained >80% for second boosters.
7	Schjoedt et al. (2023)	*Dapagliflozin Effects on Hospitalizations in Chronic Kidney Disease: DAPA-CKD Post Hoc Analysis*	CKD patients across 21 countries	4,304 adults with chronic kidney disease (2017–2020)	Multinational	Randomized Clinical Trial	Post hoc analysis of the DAPA-CKD trial. Outcomes assessed via Cox regression, Lin-Wei-Yang-Ying, and negative binomial models.	Dapagliflozin reduced first hospitalization risk (HR: 0.77) and all-cause hospitalization/death (rate ratio: 0.76). Benefits consistent regardless of diabetes status.

8	Altizani et al. (2022)	*COVID-19 Measures Reduced Pediatric Respiratory Hospitalizations in São Paulo*	Brazilian children with respiratory infections	Children <16 hospitalized for respiratory infections (2008–2021)	Brazil	Ecological Study	Ecological time-series of acute respiratory infection hospitalizations (2008–2021) in São Paulo. Predicted vs. observed admissions compared post-COVID-19.	**Hospitalizations for pediatric respiratory infections dropped below forecasts** during 2020–2021. Mortality tripled vs. pre-pandemic, with higher costs per admission.
9	Nakamura et al. (2022)	*Pathways to Reducing Hospitalizations in Older Adults: Physical, Behavioral, and Psychosocial Factors*	US adults >50 years	11,374 Health and Retirement Study participants	USA	Cross-Sectional	Generalized linear models applied to HRS data (US adults >50). Lagged exposure analysis of 62 predictors over 4 years.	Changes in physical health (e.g., cancer: HR 1.57), behavioral (e.g., exercise: HR 0.80), and psychosocial factors (e.g., volunteering: HR 0.73) predicted hospitalizations.

10	Amioka et al. (2022)	*Innovative Clinical Pathway Shortens Hospitalization for Acute Heart Failure*	ADHF patients in Japan	593 ADHF patients (2014–2019)	Japan	Retrospective	Retrospective pre-post analysis of ADHF patients. Outcomes: length of stay (LOS) and readmissions pre- vs. post-pathway.	Implementation of a clinical pathway reduced heart failure hospitalization length **[20 vs. 12 days, P<0.001]** without increasing mortality or 30-day readmissions.

11	Wood et al. (2022)	*Trends in Preventable Hospitalizations Before and After the Affordable Care Act*	US hospitalized adults	Adults 18–64 from HCUP National Inpatient Sample	USA	Cross-Sectional	Logistic regression and trend analysis of preventable hospitalization rates using HCUP data. Included ICD-9/10 codes for preventable conditions in adults aged 18–64.	**45 million preventable hospitalizations** reported between 2005–2017. Post-Affordable Care Act, preventable hospitalization rates declined from **12.0% to 10.8%** ($p<0.01$); post-Medicaid expansion: **11.5% to 10.6%** ($p<0.01$). Bacterial pneumonia rates fell from **1.5% to 0.6%** ($p<0.01$); COPD/asthma in older adults decreased from **1.9% to 1.7%** ($p=0.01$).

12	Friedrich et al. (2022)	*Impact of COVID-19 Mitigation on Asthma Hospitalizations in Brazil*	Brazilian asthma patients	Asthma patients in Brazil (2016–2020)	Brazil	Ecological	Ecological analysis of asthma admissions (Jan 2016–Dec 2020) from Brazil's Public Health Informatics Database. Absolute/relative reductions calculated pre- vs. post-pandemic.	**Asthma hospitalizations declined by 59% (ages 1–14), 37% (20–59), and 60% (≥60)** during 2020. Reductions linked to fewer respiratory viral infections amid COVID-19 restrictions.

13	Streisel et al. (2022)	*COVID-19 Stay-at-Home Orders and Trauma Hospitalization Inequities*	Trauma patients in North Carolina	70,478 trauma admissions in North Carolina	USA	Cross-Sectional	Interrupted time-series analysis of trauma admissions (2019–2020) using the North Carolina Trauma Registry. Segmented regression assessed policy impacts by race, age, and sex.	Post-lockdown, **assault-related hospitalizations rose** among Black/African American males aged 18–44. Unintentional injury admissions temporarily decreased but returned to pre-pandemic levels within months.

14	Green et al. (2022)	*Age-Based Vaccine Eligibility Reduced COVID-19 Hospitalizations in NYC*	NYC residents	Adults aged 45–84 in NYC	USA	Quasi-Experimental	Regression discontinuity design using age-specific COVID-19 hospitalization rates pre-/post-vaccine rollout (Feb–Apr 2021). Negative binomial models stratified by race/neighborhood.	**COVID-19 hospitalizations reduced by 22% (RR: 0.78, 95% CI: 0.64–0.94)** in vaccine-eligible adults aged 65–84 vs. 45–64. Estimated **721 hospitalizations prevented** in NYC.

15	Olsson Akefeldt et al. (2022)	*Rotavirus Vaccination Reduced Pediatric Gastroenteritis Hospitalizations*	Swedish pediatric patients	3,513 children with gastroenteritis	Sweden	Retrospective	Retrospective analysis of hospitalizations for gastroenteritis (2008–2018). Viral diagnostics and medical records reviewed across 10 infection seasons.	Post-rotavirus vaccination, **rotavirus gastroenteritis hospitalizations dropped by 78%** (IRR: 0.22, p<0.001). All-cause gastroenteritis rates fell by **55%** (IRR: 0.45, p<0.001). Complications (e.g., seizures) increased post-vaccination (8.8% vs. 11.4%, p<0.05).

16	Chadhuri et al. (2021)	*Machine Learning Interventions Reduce Hospitalizations in Dialysis Patients*	US hemodialysis patients	Hemodialysis patients in 108 clinics	USA	Interventio n	Quasi-experimental study comparing 54 ML-guided clinics vs. 54 controls. Paired t-tests analyzed pre-post changes in admission rates (2015–2018).	**ML-based care reduced hospitalization rates** in hemodialysis patients: Yearly admissions decreased by **0.38 per patient-year** ($p<0.001$); hospital days declined by **2.78 days per patient-year** ($p<0.001$).

17	Vogelsmeier et al. (2021)	*Missouri Initiative Reduced Nursing Home Hospital Transfers*	Long-term care residents	1,819–1,068 nursing home residents	USA	Interventio n	Six-year CMS-funded demonstration project. Descriptive analysis of transfer rates in 16 Midwest nursing homes.	**Hospital transfers declined from 2.48 to 1.89 per 1,000 resident-days** (2014–2018) in nursing homes. Leadership stability correlated with sustained reductions.

18	Imodi et al. (2020)	*Varicella Vaccination Reduced Hospitalizations in Vaccinated/Unvaccinated Cohorts*	Italian varicella patients	Varicella patients (2003–2018)	Italy	Retrospective	Retrospective analysis of Italian hospitalization records (ICD-9 code 052.X). Joinpoint regression assessed annual percent changes.	**Varicella hospitalizations fell by 80% (<1 year) and 86.7% (1–5 years)** post-vaccination. Observational data from 2003–2018 showed sustained declines across age groups.

19	Kelly et al. (2020)	*ED Navigation Reduced Hospitalizations in Medicaid High-Utilizers*	Medicaid patients	100 Medicaid-enrolled ED frequent users	USA	RCT	RCT comparing ED navigation (ED-PN) vs. usual care (UC) in 100 high-utilizers. Outcomes tracked 12 months pre-/post-intervention.	ED navigation program reduced **ED visits by 1.4 per patient** (p=0.01) and **hospitalizations by 1.0 per patient** (p=0.001) among Medicaid users. No significant change in costs.

20	Plakht et al. (2020)	*Guideline Adherence Post-AMI Lowers Mortality and Healthcare Use*	Israeli AMI patients	AMI patients at a tertiary center	Israel	Retrospective	Retrospective cohort of 8,287 AMI survivors. Adherence defined as ≥80% guideline-recommended medication fills.	Guideline-adherent post-AMI care reduced **mortality (22.0% vs. 34.2%, p<0.001)**, hospitalizations, and costs. Adherent patients had higher primary care use.

21	Zinti et al. (2020)	*Does Adherence to Quality Care Metrics Reduce Hospitalizations in Early Rheumatoid Arthritis?*	Italian RA patients	14,203 early RA patients in Lombardy	Italy	Retrospective Cohort	Retrospective analysis of 14,203 early RA patients (2006–2012). Six quality indicators (e.g., DMARD prescriptions, clinical assessments) were used to calculate adherence scores.	**Adherence to RA care standards** (e.g., early DMARDs) reduced hospitalization risk. High/moderate adherence groups had **significantly lower hospitalization rates** vs. low adherence. Long-term glucocorticoid use worsened outcomes.

22	Stopyra et al. (2020)	*HEART Pathway Safely Reduces 1-Year Hospitalizations in Acute Chest Pain*	US ED patients with chest pain	8,474 ED patients with suspected ACS	USA	Interventio n	Pre-planned analysis of 8,474 ED patients with suspected ACS. HEART Pathway stratified patients into low-risk (discharge) vs. non-low-risk (further evaluation). Outcomes tracked via health records and insurance claims.	**HEART Pathway implementation reduced 1-year hospitalizations by 7%** (p<0.001) without increasing mortality or myocardial infarction risk. Low-risk patients (30.7%) had 97.5% 1-year event-free survival.

23	Pantell et al. (2020)	*Social Needs Navigation Reduces Pediatric Hospitalizations: A Randomized Trial*	Low-income families in California	1,809 caregivers of pediatric patients	USA	RCT	RCT comparing in-person navigation (n=637) vs. active control (n=663) in 1,809 caregivers. Social needs addressed via tailored resources and follow-ups.	**In-person social needs navigation reduced pediatric hospitalizations by 69%** (p<0.001) but did not affect ED visits. No cost differences observed.

24	Szarek et al. (2019)	*Alirocumab Reduces Hospitalizations in Post-Acute Coronary Syndrome Patients*	High-risk cardiovascular patients	17,355 post-ACS patients	Multinational	RCT	Pre-specified analysis of ODYSSEY OUTCOMES trial (n=17,355). Semi-parametric models assessed hospitalization/death rates; beta regression analyzed DAOH.	**Alirocumab reduced total hospitalizations by 15.6 per 1,000 patient-years** and increased days alive and out of hospital (DAOH). Mortality risk also decreased.

25	Luthra et al. (2019)	*Endoscopic Interventions Reduce Readmissions in Biliary Pancreatitis During Pregnancy*	Pregnant women with ABP	7,787 ABP admissions in pregnant women	USA	Cross-Sectional	Propensity-matched analysis of 7,787 pregnant women with acute biliary pancreatitis (ABP) using US Nationwide Readmissions Database (2011–2014).	**ERCP and cholecystectomy during pregnancy reduced 30-day readmissions** ($p<0.01$). Weekend admissions and prolonged stays increased readmission risk.

26	Singh et al. (2019)	*PAP Therapy Lowers COPD-Related Hospitalizations in Overlap Syndrome*	Medicare beneficiaries with COPD-OSA	319 COPD-OSA patients	USA	Retrospective Cohort	Retrospective cohort of 319 Medicare beneficiaries with COPD-OSA. Outcomes compared 1 year pre- and post-PAP initiation.	**PAP therapy in COPD-OSA overlap reduced COPD-related hospitalizations** but not all-cause admissions or ED visits. Benefits greater in older, comorbid patients.

27	Rahaingomanana et al. (2019)	*Rotavirus Vaccination Cuts Diarrhea Hospitalizations in Madagascar*	Malagasy children with diarrhea	19,619 children <5 years	Madagascar	Cross-Sectional	Cross-sectional analysis of 19,619 pediatric gastroenteritis admissions (2010–2016). WHO-supported surveillance identified rotavirus genotypes pre-/post-vaccine.	**Rotavirus vaccination reduced diarrhea hospitalizations by 55%** (IRR: 0.45, p<0.001). Rotavirus-positive stool samples declined from 56% to 12%.

28	Hung et al. (2018)	*Periodontal Care Lowers Cardiovascular Hospitalizations in Dialysis Patients*	Dialysis patients with periodontal disease	3,613 hemodialysis patients	Multinational	Retrospective Cohort	Retrospective cohort of 3,613 hemodialysis patients (1998–2011). Propensity-matched analysis using national insurance data.	**Intensive periodontal therapy reduced CV hospitalization risk by 22%** (HR: 0.78, $p<0.001$) and all-cause mortality in hemodialysis patients.

29	Wright et al. (2018)	*Early Palliative Care Reduces End-of-Life Hospitalizations in Cancer*	Terminal cancer patients in Western Australia	28,331 cancer decedents	Australia	Retrospective	Population-based study of 28,331 cancer decedents (2001–2011). Linked administrative data assessed CPC timing and outcomes.	**Early community palliative care (CPC) reduced unplanned hospitalizations** (1.4 vs. 1.7 admissions, $p<0.01$) and costs ($12,976 vs. $13,959) in terminal cancer patients.

30	Halterman et al. (2018)	*Telemedicine Program Lowers Asthma Hospitalizations in Schoolchildren*	Urban schoolchildren with asthma	400 children with persistent asthma	USA	RCT	RCT of 400 children (2012–2016). SB-TEAM combined telemedicine visits and supervised inhaler use vs. usual care.	**School-based telemedicine program reduced asthma hospitalizations** ($p < 0.01$) and increased symptom-free days. No effect on ED visits.
